# TGFBI promotes proliferation and epithelial–mesenchymal transition in renal cell carcinoma through PI3K/AKT/mTOR/HIF-1α pathway

**DOI:** 10.1186/s12935-024-03454-7

**Published:** 2024-07-27

**Authors:** Shanzhi Zhan, Xiaojie Bai, Yiqiao Zhao, Kuerban Tuoheti, Zuhaer Yisha, Yingtong Zuo, Peixiang Lu, Tongzu Liu

**Affiliations:** Department of Urology, Zhongnan Hospital of Wuhan University, 169 Donghu Road, Wuhan, 430071 China

**Keywords:** TGFBI, Renal cell carcinoma, Migration, Invasion, Epithelial–mesenchymal transition, PI3K/AKT/mTOR/HIF-1α pathway

## Abstract

**Background:**

Renal cell carcinoma (RCC) is presently recognized as the most prevalent kidney tumor. However, the role and underlying mechanism of action of the conversion factor-inducible protein (TGFBI), an extracellular matrix protein, in RCC remain poorly understood.

**Methods:**

In this study, we employed Western blot, quantitative real-time polymerase chain reaction (qRT-PCR), and immunohistochemistry techniques to assess the expression of TGFBI in RCC tissues or cells. Furthermore, we analyzed the proliferation and migration of RCC cells using CCK8, cloning, scratching, and migration assays. Additionally, we examined apoptosis and cell cycle progression through flow cytometry, analysis. Lastly, we employed gene set enrichment analysis (GSEA) to investigate the biological processes associated with TGFBI, which were subsequently validated.

**Results:**

The findings indicate that TGFBI exhibits significantly elevated expression levels in both renal cell carcinoma (RCC) tissues and cells. Furthermore, the knockdown of TGFBI in SiRNA transfected cells resulted in the inhibition of RCC cell proliferation, migration, invasiveness, apoptosis, and alteration of the cell cycle. Additionally, TGFBI was found to impede the epithelial-mesenchymal transition (EMT) process in RCC cells. Bioinformatics analysis suggests that TGFBI may exert its influence on various biological processes in RCC through the tumor immune microenvironment. Moreover, our study demonstrates that TGFBI promotes RCC progression by activating the PI3K/AKT/mTOR/HIF-1α.

**Conclusions:**

Our research indicates that TGFBI exhibits high expression in RCC and facilitate RCC progression and metastasis through various molecular mechanisms. Hence, TGFBI has the potential to be a novel therapeutic target for the diagnosis and treatment of RCC in the future.

**Supplementary Information:**

The online version contains supplementary material available at 10.1186/s12935-024-03454-7.

## Introduction

Renal cancer is one of the top 10 most common tumors in the world. According to recent statistics from the International Agency for Research on Cancer (IARC) under the World Health Organization (WHO), about 430,000 people were diagnosed with kidney cancer and 180,000 more died from the disease in 2020. Among them, renal cell carcinoma accounts for more than 90% of the total number of kidney cancers, and the most common type is clear cell carcinoma of the kidney [1]. At present, the main treatment for renal cell carcinoma remains surgical resection. However, for some patients with inoperable advanced metastasis and postoperative adjuvant targeted chemotherapy, there are currently not many available drugs. Although some targeted molecules commonly used in clinical practice, such as mTOR inhibitors (temsirolimus) and VEGF (bevacizumab), have been proven to be effective in RCC [2, 3], the emergence of drug resistance still leads to an unsatisfactory prognosis of RCC [4]. Therefore, it is of great significance for diagnosis and treatment to find key biomarkers of RCC and comprehensively understand its molecular mechanism.

Transforming growth factor β inducible protein (TGFBI) is a cellular matrix protein, also known as BIGH3, that is involved in a variety of biological processes during embryonic development and disease development, such as cell adhesion and bone formation [5]. It was first found to be highly expressed in TGF-β-responsive prostate and lung cancers [6] ,later studies have suggested that there is also high expression in the extracellular matrix of the cornea [7]. Previous studies have proposed that TGFBI has both tumor suppressor and tumor promoting effects, and with the deepening of research, it has been confirmed that TGFBI plays an important role in promoting tumor growth in diagnosed tumor patients, and may have an inhibitory effect on tumor growth in the early stage of tumor initiation [5]. TGFBI has been reported to play an important role in prostate, ovarian, breast, colon, glioblastoma, and other tumors [8–12]. Recent studies have also suggested that TGFBI may promote tumor growth and drug resistance by affecting the tumor microenvironment of pancreatic cancer [13]. However, there are still few studies on the role of TGFBI in renal cell carcinoma, and the biological process of its expression and participation in renal cell carcinoma is still unclear, which is worthy of further exploration.

In this study, we experimentally verified that TGFBI is indeed highly expressed in renal cell carcinoma, and then further verified the promotion of TGFBI on the proliferation, migration and invasion of RCC cells in two selected renal cancer cell lines, and found that it may play a role by activating the PI3K/AKT/mTOR/HIF-1α signaling pathway.

## Materials and methods

### Bioinformatics parts

Source data for this part were acquired from The Cancer Genome Atlas (TCGA) datasets and Clinical Proteomic Tumor Analysis Consortium (CPTAC) portal. To engage differentially expression, gene correlation and drug prediction, we applied for R software (v 4.3.1). Gene Set Enrichment Analysis (GSEA) software (v 4.3.2) was utilized to visualize the results. Online sites were also used for deeper analyzation, single cell profiles were obtained from former research [14] and visualized by Single Cell Portal (SCP) (https://singlecell.broadinstitute.org/single_cell).lymphocyte correspondence data was attained from TISIDB [15]. Molecular docking was engaged by CB Dock2 [16].

### Patients and tissue samples

We obtained 10 cases of kidney cancer cells and adjacent normal tissues from the Department of Urology, Zhongnan Hospital, Wuhan University. All tissue specimens are flash frozen in liquid nitrogen immediately after excision and then stored at -80 °C for subsequent analysis validation. The pathological diagnosis of RCC in these patients was diagnosed and reviewed by 2 senior pathologists. All experimental procedures were approved by the Ethics Committee of Zhongnan Hospital of Wuhan University.

### Cell lines and cell culture

Human renal cell carcinoma cell lines (786-0, ACHN, 769-P, and Caki-1) and normal human primary renal tubular epithelial cells (HK-2) were purchased from Stem Cell Bank, Chinese Academy of Sciences in Shanghai, China.786-0 and 769-P cells were cultured in RPMI 1640 medium (Gibco, China), and ACHN cells were cultured in MEM medium (Gibco, China) and Caki-1 cells were cultured in McCoy’s 5 A medium (Gibco, China) supplemented with 10% fetal bovine serum (Gibco, China). All cells were cultured at 37 °C, 5% CO2 in a humidified atmosphere.

### Cell viability assays

Cell viability was assessed using the Cell Counting Kit-8 (CCK-8) (Sangon Biotech, Shanghai, China) following the manufacturer’s instructions. We seeded kidney cancer cells (1000 cells/well) in 96-well plates, added 10 µl of CCK-8 solution to each well, and incubated at 37 °C for 2 h in the dark. Finally, the absorbance was measured by microplate reader at 450 nm at 0 h, 24 h, 48 h, 72 h and 96 h, respectively, and the cell proliferation curves were plotted according to the corresponding data.

### Colony formation assay

First, after successful transfection, well-growing cells are seeded in a 6-well dish (1000 cells/well) and incubated for 10–14 days. Wash 2 times with PBS and fix with 500 ul of 4% paraformaldehyde for 20 min. Then, stain with 500ul of 0.1% crystal violet for 20 min, then slowly wash off the staining solution with running water and air dry. Finally, count more than 50 colonies using an ImageJ cell counter.

### Wound healing assay

Wound healing assays were used to assess the effect on cell migration capacity. After successful transfection, well-grown cells are seeded in six-well plates and cultured to form a confluent monolayer. Scrape the wound with a sterile 200 µl pipette tip. Wash three times with PBS to remove debris and then add FBS-free medium. Observe the spread of wound healing at 0 h, 24 h and take pictures under the microscope. Calculate the surface area of the wound using ImageJ. Cell migration results are shown as the proportion of the corresponding control group.

### Transwell assay

Cell invasion was assessed by the Transwell method, 100 µl (about 50,000 cells) of serum-free medium resuspension was added to the upper chamber chamber, 600 µl of medium containing 10% fetal bovine serum was added to the lower chamber, incubated at 37 °C for 12–18 h and then removed, and the upper chamber was washed and air-dried with PBS. Place the air-dried upper chamber into a new 24-well plate, immerse in 600 µl of 4% paraformaldehyde, fix for 20 min, and wash 1–2 times in PBS. Place the chamber into a new clean well, add 600 µl of 0.1% crystal violet, wash again 2 times after staining for 20 min, and dry thoroughly. Finally, 5 random fields per well were observed and counted under the microscope.

### Real‑time quantitative reverse transcription PCR (qRT‑PCR) analysis

The RaPure Total RNA Micro Kit (Magen, China) was used to extract total RNA from various kidney cancer cells. RNA was quantified at 260 nm/280 nm using a NanoDrop2000 spectrophotometer (Thermo Fisher Scientific, Waltham, MA, USA). Total RNA was reverse transcribed to cDNA using ABScript II RT Master Mix (ABclonal, Wuhan, China). qRT-PCR was performed on a Bio-Rad (Hercules, CA, USA) CFX96 system to determine the mRNA level of the gene of interest based on the SYBR green fluorescence level. The primers used and their sequences are shown in Table S1. The 2−∆∆CT method was used combined with ACTB as a control to evaluate the relative expression level of mRNA of each target gene.

### Western blot analysis

Total protein was extracted by thorough lysis of tissues or cells (all from Beyotime, Shanghai, China) with RIPA containing 1% protease inhibitor, separated by SDS-PAGE electrophoresis, and transferred to a polyvinylidene fluoride (PVDF) membrane (Millipore, cat# IPVH00010, Shanghai, China). After blocking with 5% skim milk for 2 h at room temperature, first incubate overnight at 4 °C with primary antibody (Table S2) and then with secondary antibody goat anti-rabbit IgG (Table S3) for 1 h at room temperature. The bands on the membrane were exposed on the Tanon-5200 ECL imager (Tanon, Shanghai, China) and visualized with an enhanced chemiluminescence kit (Thermo Fisher Scientific, Waltham, MA, USA). Sc79 (AKT activator) and L Y294002 (PI3K inhibitor) were acquired from MedChemExpress (MedChemExpress, Monmouth Junction, NJ, USA).

### Immunohistochemical (IHC)

The obtained human kidney cancer tissue and paracancerous tissue were dewaxed with formalin-fixed and paraffin-embedded tissue sections. Endogenous peroxidase activity is then inhibited with H2O2. Add the specified primary antibody (Table S2) and secondary antibody (Table S3) to the sections according to the recommended protocol provided by the manufacturer. All slides were examined under a 400x inverted microscope.

### Cell transfection

Two siRNAs targeting TGFBI (siRNA-1 sense: 5’-GAUAAGGUCAUCUCUCACCATT-3’; siRNA-2 sense: 5’-CUUGAAGUCAGCUAUGUGUTT-3’) were constructed by Genepharma in Suzhou, China. Plasmid vectors (Fenghui Biotechnology Co., Ltd, Changsha, China) were used for the construction of overexpression. GP-transfect-Mate (Genepharma, Suzhou, China) was used for transient transfection. Forty-eight hours after transfection, the function of siRNA was detected by Western blotting and qRT-PCR.

### Cell-apoptosis and cell-cycle analysis

Apoptosis and cycle were assessed by flow cytometry (FCM) analysis. Different cells were collected for 48 h and then washed with ice-cold phosphate-buffered saline (PBS) and stained with Cell Cycle and Apoptosis Analysis Kit (Beyotime, Shanghai, China). Stained cells were detected with flow cytometry (Beckman Coulter, USA).

### Statistical analysis

Data are presented as the mean ± standard errors of at least three independent experiments. Statistical analysis was done by the software GraphPad Prism 8.0.2. The quantitative data were analyzed using Student’s t-tests or one-way ANOVA, with *p* < 0.05 were considered statistically significant.

## Results

### The expression of TGFBI in RCC and corresponding normal tissues and its role in survival and clinical applications

**Fig. 1:**
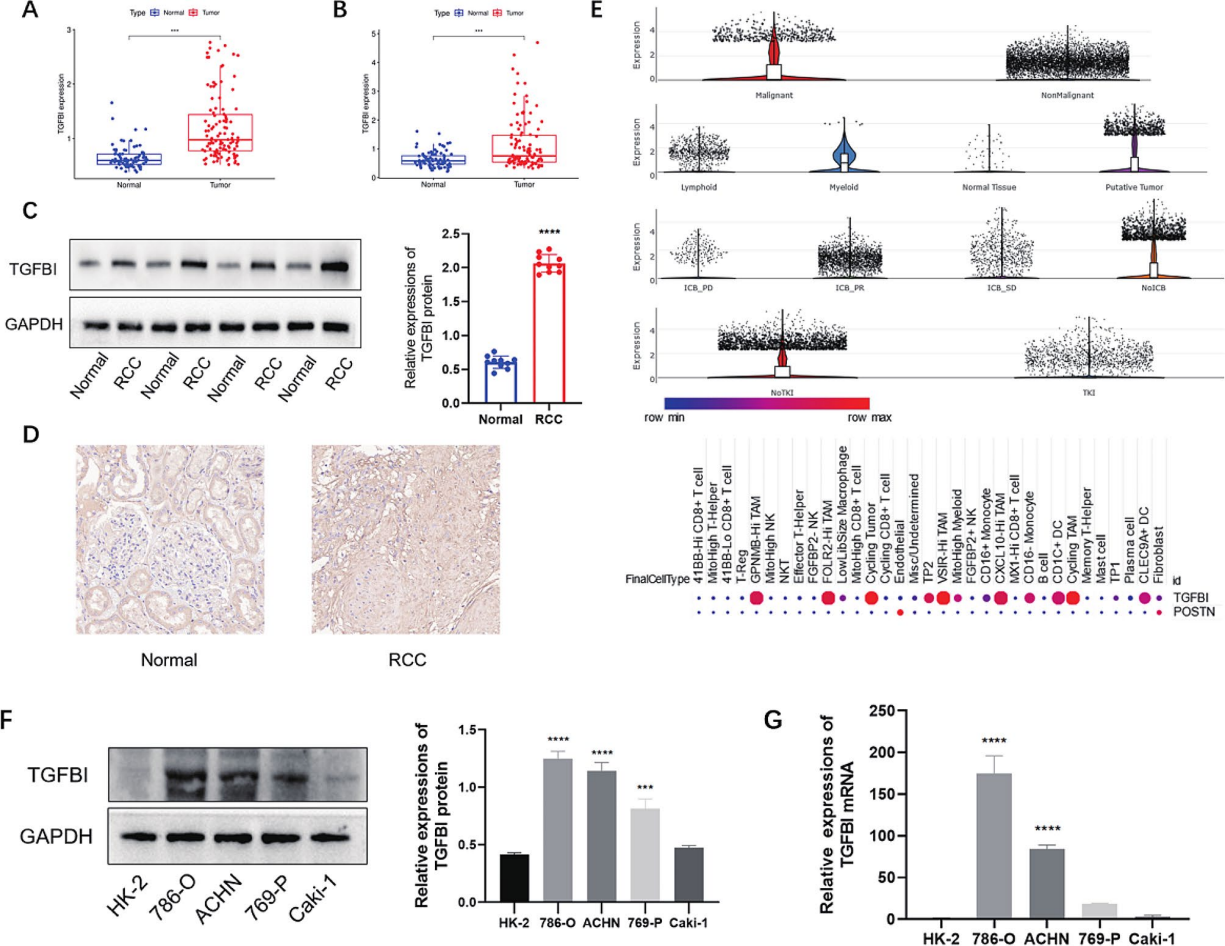
Differentially expression analyses of TGFBI total protein level **(A)** and phosphoprotein level **(B)** between normal and tumor tissues via data acquired from CPTAC. TGFBI protein expression levels were significantly upregulated in RCC tissues compared to adjacent normal tissues. ***p < 0.001 compared with normal tissues **(C-D)**. Explorations of single cell data revealed higher expression of TGFBI in malignant group and putative tumor group, patients who receive TKI and ICB treatment had lower expression of TGFBI. TAMs and cycling TAM were found to accompany with significantly stronger TGFBI expression compared to other cells **(E)**. TGFBI expression in various RCC cells. **(F)** Western blot analysis of TGFBI; **(G)** mRNA expressions of TGFBI in different cells. *** *p* < 0.001, and **** *p* < 0.0001 compared with HK-2.

The TCGA and CPTAC databases were used in this study to characterize the differences in TGFBI expression in RCC and normal samples. Despite that the expression of TGFBI mRNA level was analyzed and validated in our previous research [17], the expression level of protein and phosphoprotein level of TGFBI still remained unknown, thereby we analyzed the data obtained from CPTAC and discovered a higher expression of TGFBI in KIRC at both total (Fig. 1A) and phosphoprotein (Fig. 1B) levels compared to their normal counterparts. We analyzed the expression of TGFBI in tumor tissues and corresponding normal tissue specimens from RCC patients using western blot. TGFBI was significantly upregulated in RCC tissues compared with adjacent normal tissues (Fig. 1C). The expression of TGFBI in RCC patients was then analyzed by IHC (Fig. 1D), which was similar to the western blot results. By searching SCP, we further verified that TGFBI mainly residents in malignant cell and putative tumor cells, it also showed that patients who received TKI treatment had lower TGFBI level, however, when comparing its profiles in ICB responders and ICB non responders, there was no notable difference. Intriguingly, TGFBI presented remarkably higher expression in tumor associated macrophages (TAMs) comparing to other cell types, which echoed the results we obtained above(Figure 1E). To investigate the expression of TGFBI in RCC, we selected RCC cells (786-O, ACHN, 768-P, and Caki-1) for analysis and used a normal renal tubular epithelial cell line (HK-2) as a control. By Western blot, it was shown that TGFBI was overexpressed in RCC cells compared with HK-2 cells (Fig. 1F), meanwhile, we also verified that its expression at the RNA level was basically consistent with that at the protein level (Fig. 1G). By these results, it was suggested that the abnormal expression level of TGFBI might be related to RCC.

### Knocking Down TGFBI inhibits cell proliferation and migration

**Fig. 2:**
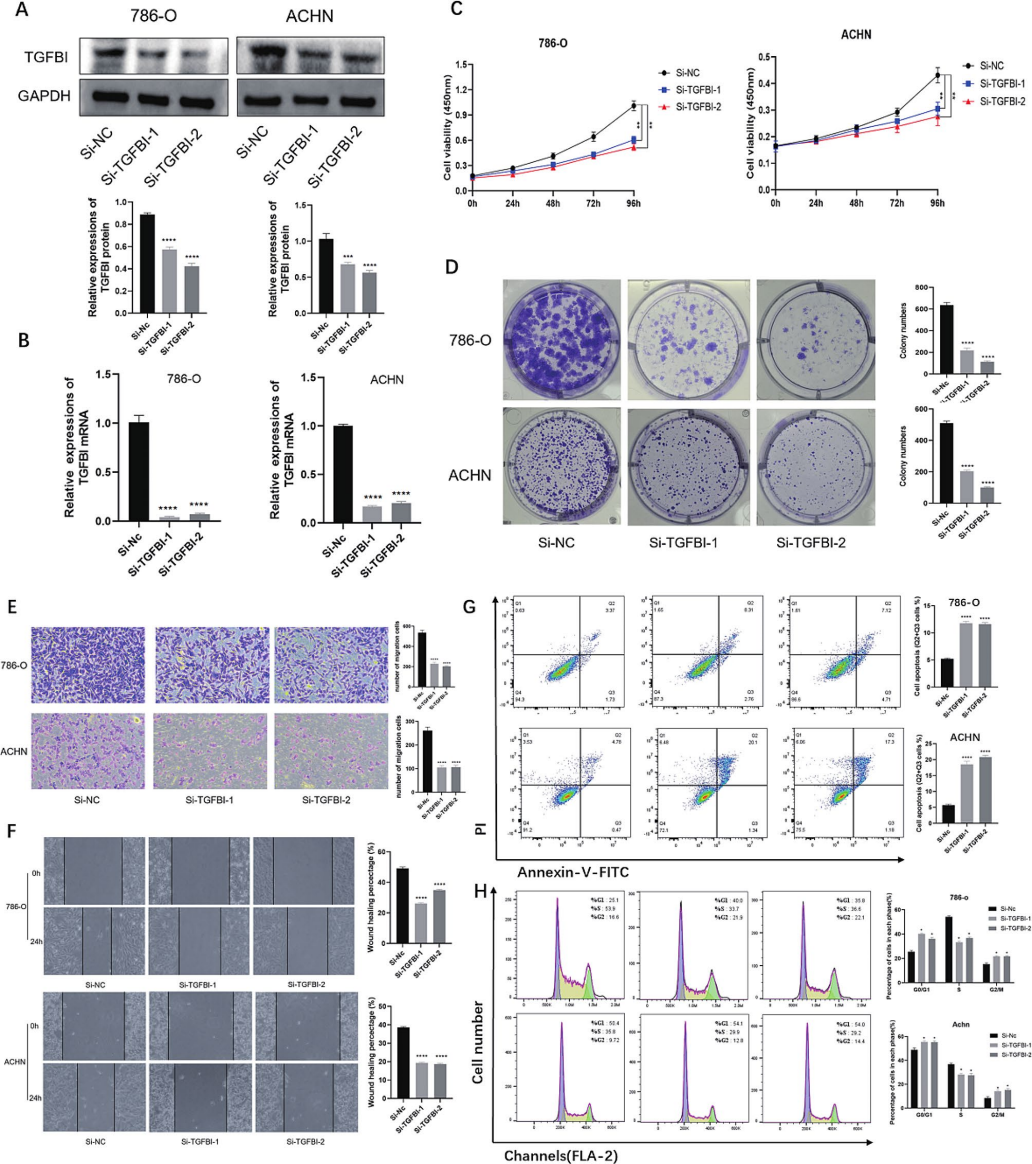
**(A-B)** Protein and RNA expression levels after knockdown of TGFBI in 786-O and ACHN cells. **(C)** The proliferation of cells in the TGFBI knockdown group and the control group at 24, 48, 72 and 96 h were detected by CCK-8 method in 786-O and ACHN cells. (D) Colony formation with TGFBI knockdown in 786-O and ACHN cells. **(E-F)** The migration and invasion of cells in the TGFBI knockdown group and the control group were detected by wound healing and transwell in 786-O and ACHN cells. **(G)** Distribution of apoptosis in TGFBI knockdown group and control group in 786-O and ACHN cells. **(H)** Proportion of each cellular phase in the TGFBI knockdown group and the control group in 786-O and ACHN cells. Data are shown as mean ± SD of three independent experiments, * *p* < 0.05, ** *p* < 0.01, *** *p* < 0.001, and **** *p* < 0.0001 compared with Si-NC.

We chose two kinds of cells, 786-O and ACHN, for the study, and transfected Si-TGFBI into the cells to knock down the expression of TGFBI gene, so as to further understand its function in biological process. The knockdown efficiency was verified by Western blot and qRT-PCR, respectively, and the expression of TGFBI in renal cancer cells after transfection with siRNA was significantly decreased compared with that in normal renal tubular epithelial cells. (Fig. 2A-B)

After verifying the knockdown of TGFBI expression, we performed CCK-8 to detect cell proliferation in renal tumor cells after knockdown of TGFBI. After transfection of Si-TGFBI in ACHN and 786-O cells, their cell proliferation was significantly lower than that of the normal group (Fig. 2C). Then, as shown by clone formation assay, knockdown of TGFBI significantly inhibited the clone formation ability of 786-O and ACHN (Fig. 2D). To further understand the effect of TGFBI on the migration and invasion profiles of RCC cells, we assessed whether TGFBI affects the migration and invasion of renal tumor cells by wound healing assay and transwell assay. Our results showed that the migration and invasion abilities of ACHN and 786-O were significantly attenuated after knockdown of TGFBI (Fig. 2E-F). All these results indicated that TGFBI promoted the migration and invasion of RCC cells.

### Knocking Down TGFBI promotes 786-O and ACHN apoptosis and cell cycle arrest at the G0/G1 and G2/M phase

Annexin V-FITC-labeled apoptotic cells were classified into four states: normal living cells, early apoptotic cells, mid-late apoptotic and necrotic cells and mechanically damaged cells. In this study, apoptosis of 786-O cells and ACHN cells was significantly increased after knockdown of TGFBI (Fig. 2G). The cell cycle changes were further evaluated using flow cytometry as well. The results showed that 786-O cells and ACHN cells showed a significant increase in the proportion of G0/G1 phase and G2/M phase cells and a significant decrease in the proportion of S phase cells in the cells after knockdown of TGFBI (Fig. 2F).

### TGFBI overexpression increases cell proliferation and migration in RCC

**Fig. 3:**
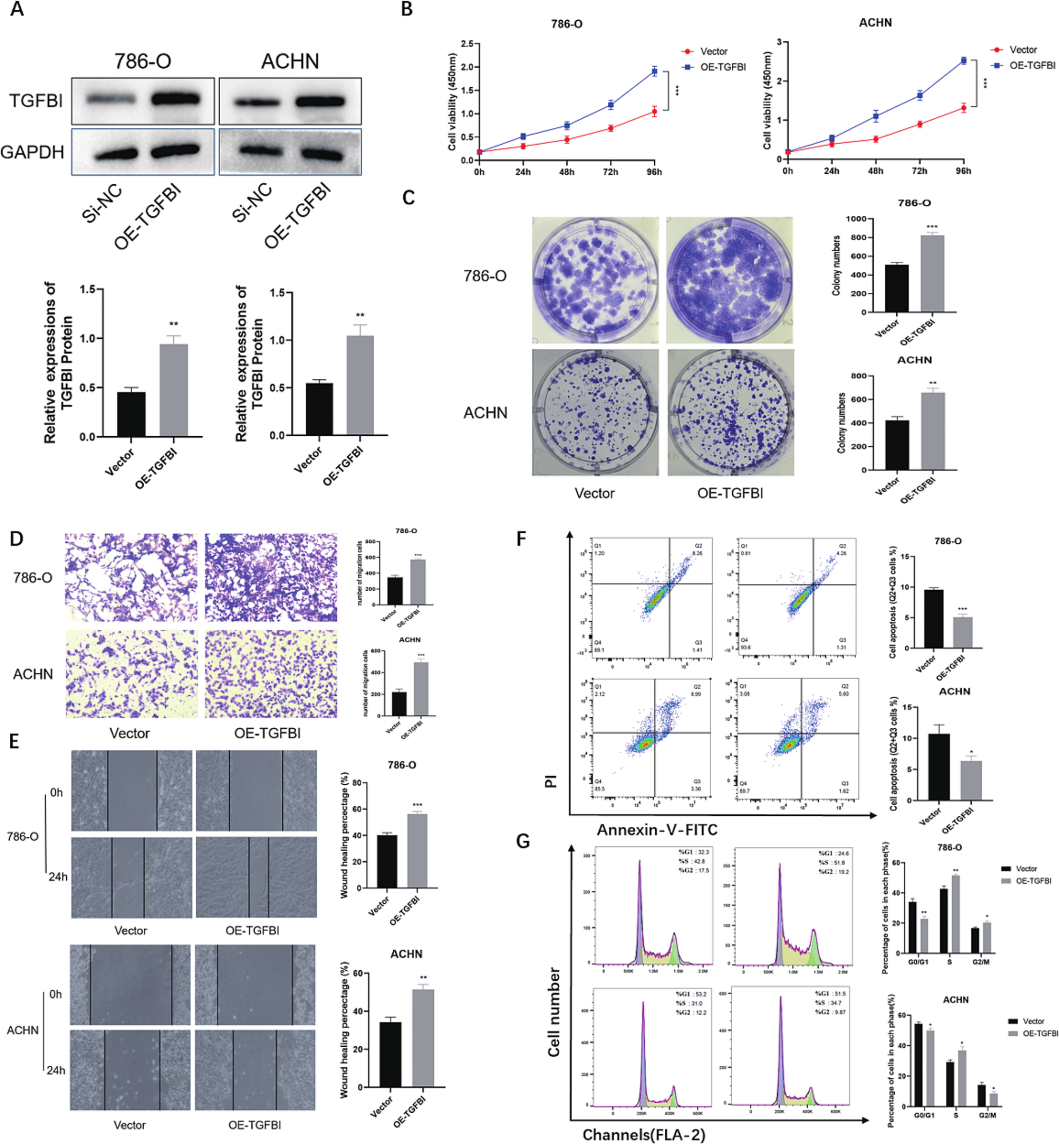
**(A)** Protein expression levels after overexpression of TGFBI in 786-O and ACHN cells. **(C)** The proliferation of cells in the TGFBI overexpression group and the vector group at 24, 48, 72 and 96 h were detected by CCK-8 method in 786-O and ACHN cells. **(D)** Colony formation with TGFBI overexpression in 786-O and ACHN cells. **(E-F)** The migration and invasion of cells in the TGFBI overexpression group and the vector group were detected by wound healing and transwell in 786-O and ACHN cells. **(G)** Distribution of apoptosis in TGFBI overexpression group and vector group in 786-O and ACHN cells. **(H)** Proportion of each cellular phase in the TGFBI overexpression group and the vector group in 786-O and ACHN cells.Data are shown as mean ± SD of three independent experiments, * *p* < 0.05, ** *p* < 0.01 and *** *p* < 0.001 compared with Vector

In the following experiments, we upregulated the expression of TGFBI in RCC cells. Overexpression efficiency was verified using western blot (Fig. 3A). We performed CCK-8 and colony formation assays to show that TGFBI overexpression enhanced cell proliferation and colony formation ability (Fig. 3B-C). Wound healing experiments and Transwell migration analysis jointly demonstrated that TGFBI overexpression significantly increased the migration ability of RCC cells (Fig. 3D-E). Moreover, Overexpression of TGFBI significantly reduced apoptosis in 786-O and ACHN cells by flow cytometry, which was beneficial to tumor progression (Fig. 3F). At the same time, the results showed that 786-O cells and ACHN cells showed a significant increase in the proportion of S phase cells and a significant decrease in the proportion of G0/G1 phase cells in the cells after overexpression of TGFBI (Fig. 3G).

### TGFBI regulates the expression of epithelial-mesenchymal transition (EMT) markers in renal cancer cells

**Fig. 4:**
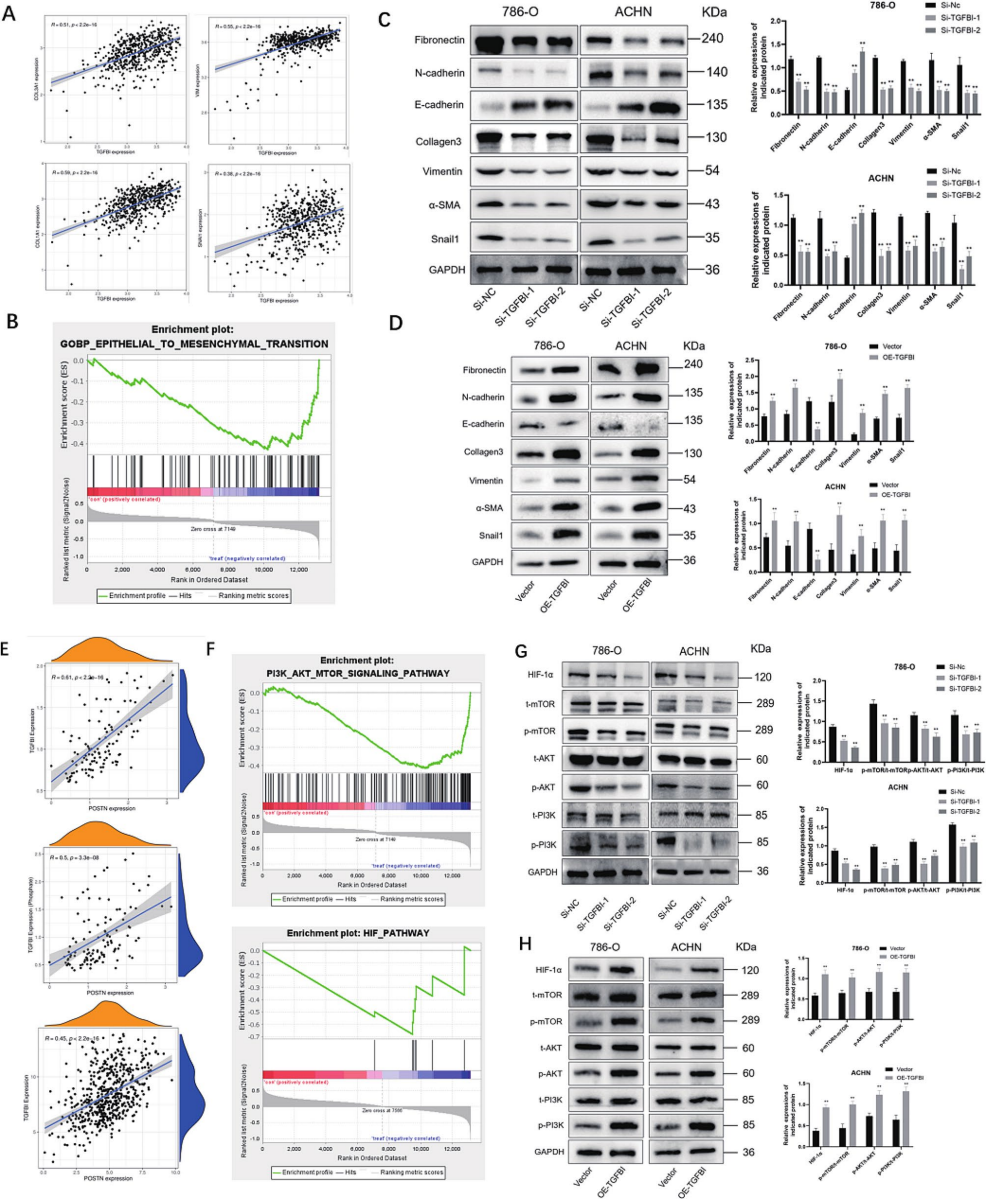
TGFBI is closely related to the transformation of epithelial-mesenchymal (EMT) processes in RCC cells. **(A)** Correlation analysis between TGFBI and some EMT-related molecules. **(B)** GSEA analysis showed that the epithelial-mesenchymal transition (EMT) process was enriched. **(C-D)** The expression levels of EMT-related molecules were analyzed by western blotting. TGFBI regulates the PI3K/AKT/mTOR/HIF-1α signalling pathway. **(E)** Correlation analyses of TGFBI and POSTN in RNA, total protein and phosphoprotein level from top to bottom. **(F)** GSEA analysis showed enrichment in PI3K/AKT/mTOR and HIF-1α signaling pathway. **(G-H)** Protein levels of the PI3K/AKT/mTOR and HIF-1α signal pathway proteins (p-PI3K, PI3K, p-AKT, AKT, p-mTOR and mTOR) were determined by western blot after knocking down TGFBI. Data are shown as mean ± SD. ** *p* < 0.01 compared with controls

Previous studies have shown that EMT plays a crucial role in the development of kidney tumor cells [18]. In our study, we first analyzed the relationship between some EMT-related molecules (e.g., COL1A1, COL3A1, Vimentin and Snail1) and TGFBI expression by bioinformatics, and found that the expression of these molecules were all positively correlated with the expression of TGFBI (Fig. 4A). In addition, we found by GSEA analysis that in EMT-related pathways were highly enriched in patients with high TGFBI expression (Fig. 4B). We confirmed in further experiments that knockdown of TGFBI significantly reduced the levels of Fibronectin, N- cadherin, Vimentin, Collagen3, α-SMA and Snail1 and increased the level of E-cadherin in both 786-O and ACHN cells (Fig. 4C). Similarly, we also examined the changes in EMT-related molecules after TFGBI overexpression. upregulation of TGFBI significantly increased the levels of Fibronectin, N- cadherin, Vimentin, Collagen3, α-SMA, Snail1 and reduced the level of E-cadherin in both 786-O and ACHN cells (Fig. 4D). All of the above analysis results indicated that TGFBI was involved in the EMT process of renal cancer cells.

### TGFBI plays a key role in the activation of PI3K/AKT/mTOR/ HIF‑1α signaling pathway in renal cancer cells

Previous literature has reported a close relationship between POSTN and TGFBI [19–22], We analyzed the correlation between TGFBI and POSTN at RNA, total protein, and phosphoprotein levels and found a positive correlation between the expression of the two (Fig. 4E), whereas previous studies have been performed on POSTN indicating the importance of the AKT/mTOR pathway in RCC [23]. To understand which signaling pathways TGFBI was involved in, we performed GSEA analysis using a bioinformatics approach and found that knockdown of TGFBI significantly inhibited the PI3K/AKT/mTOR and HIF-1 signaling pathways (Fig. 4F). Hypoxia-inducible factor-1α (HIF-1α) is a core factor of the HIF-1 signaling pathway and has been reported to play an important role in various biological behaviors of renal cell carcinoma [24, 25]. Therefore, we tried to find the relationship between TGFBI expression and HIF-1α expression, and we found a significant decrease in HIF-1α expression after knockdown of TGFBI in 786-O and ACHN by subsequent Western bolt. The PI3K/AKT/mTOR signaling pathway has been reported to have a regulatory effect on HIF-1α protein expression in previous literature, [24] and combined with our GSEA analysis, we verified this and found that knockdown of TGFBI resulted in a significant decrease in phosphorylated PI3K, AKT, and mTOR, but did not affect total protein expression (Fig. 4G). Similarly, overexpression of TGFBI significantly increased the level of phosphorylated PI3K, AKT, and mTOR, but did not affect total protein expression (Fig. 4H). In summary, we concluded that TGFBI activates the PI3K/AKT/mTOR/HIF-1α pathway in RCC cells.

### SC79 reverses the effect of TGFBI downregulation in renal cancer cells

**Fig. 5:**
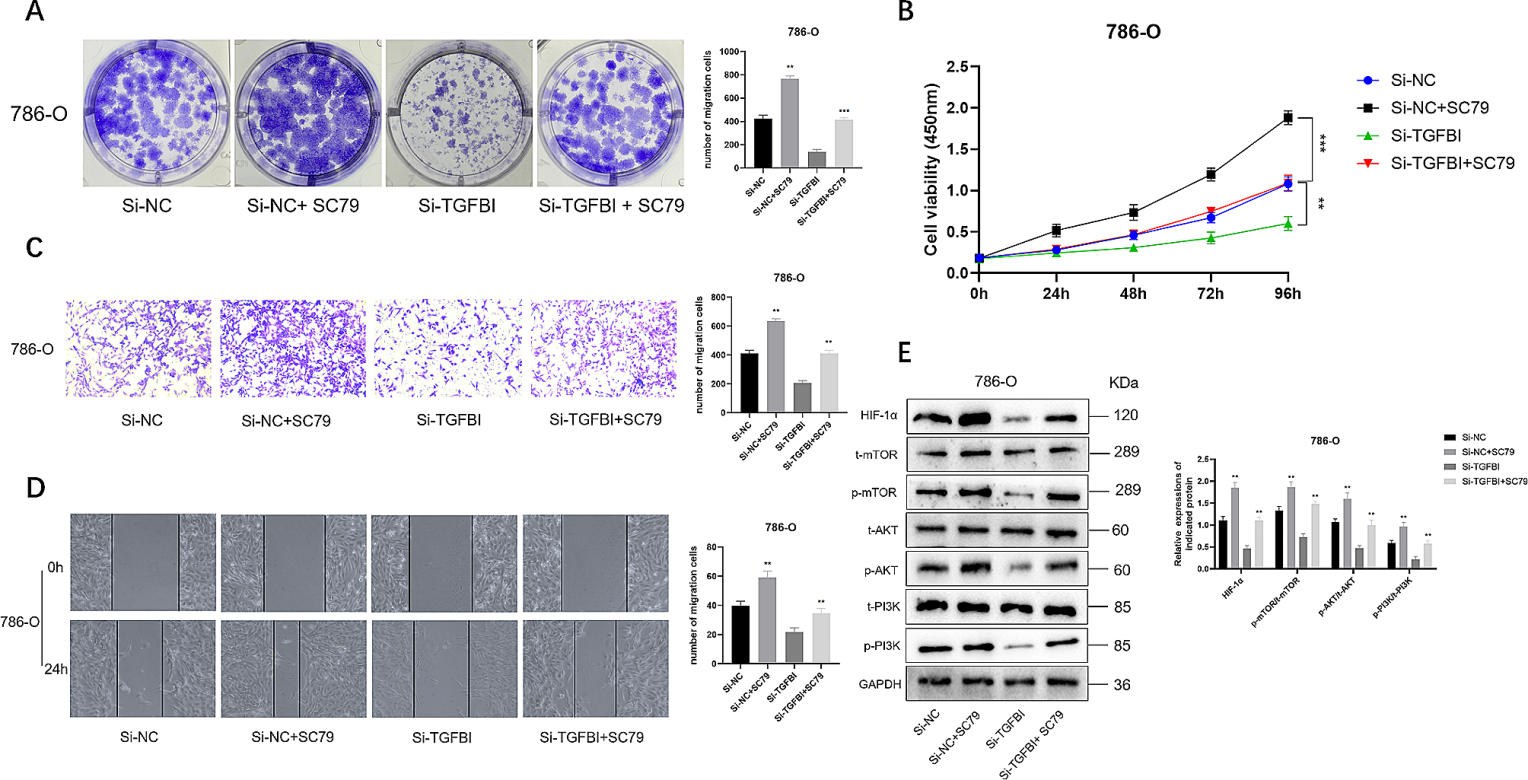
SC79 recovers the effect of TGFBI knockdown in renal cancer cells. **(A)** Colony formation in the Si-NC, Si-NC + SC79, Si-TGFBI, Si-TGFBI + SC79 group in 786-O cells. **(B)** The proliferation of cells in the Si-NC, Si-NC + SC79, Si-TGFBI, Si-TGFBI + SC79 group at 24, 48, 72 and 96 h were detected by CCK-8 method in 786-O cells. **(C-D)** The migration and invasion of cells in the Si-NC, Si-NC + SC79, Si-TGFBI, Si-TGFBI + SC79 group were detected by wound healing and transwell in 786-O cells. **(E)** Western blotting analysis of the PI3K/AKT/mToR/HIF-1α signalling pathway-related protein expression in renal cancer cells. Data are shown as mean ± SD. ** *p* < 0.01, *** *p* < 0.001 compared with controls

To further confirm the role of the PI3K/AKT/mTOR/HIF-1α signaling pathway in TGFBI-mediated tumor promotion, we used the AKT activator SC79 to treat 786-O cells transfected with TGFBI siRNA. The results showed that SC79 could reverse the reduced cell proliferation and colony formation ability caused by TGFBI knockdown (Fig. 5A-B). Similarly, SC79 also reversed the decrease in cell migration and invasion abilities caused by TGFBI downregulation (Fig. 5C-D). In addition, SC79 treatment also increased the phosphorylation levels of AKT and mTOR and the levels of HIF-1α in TGFBI-downregulated cells (Fig. 5E).

### LY294002 recovers the effects of TGFBI upregulation in renal cancer cells

**Fig. 6:**
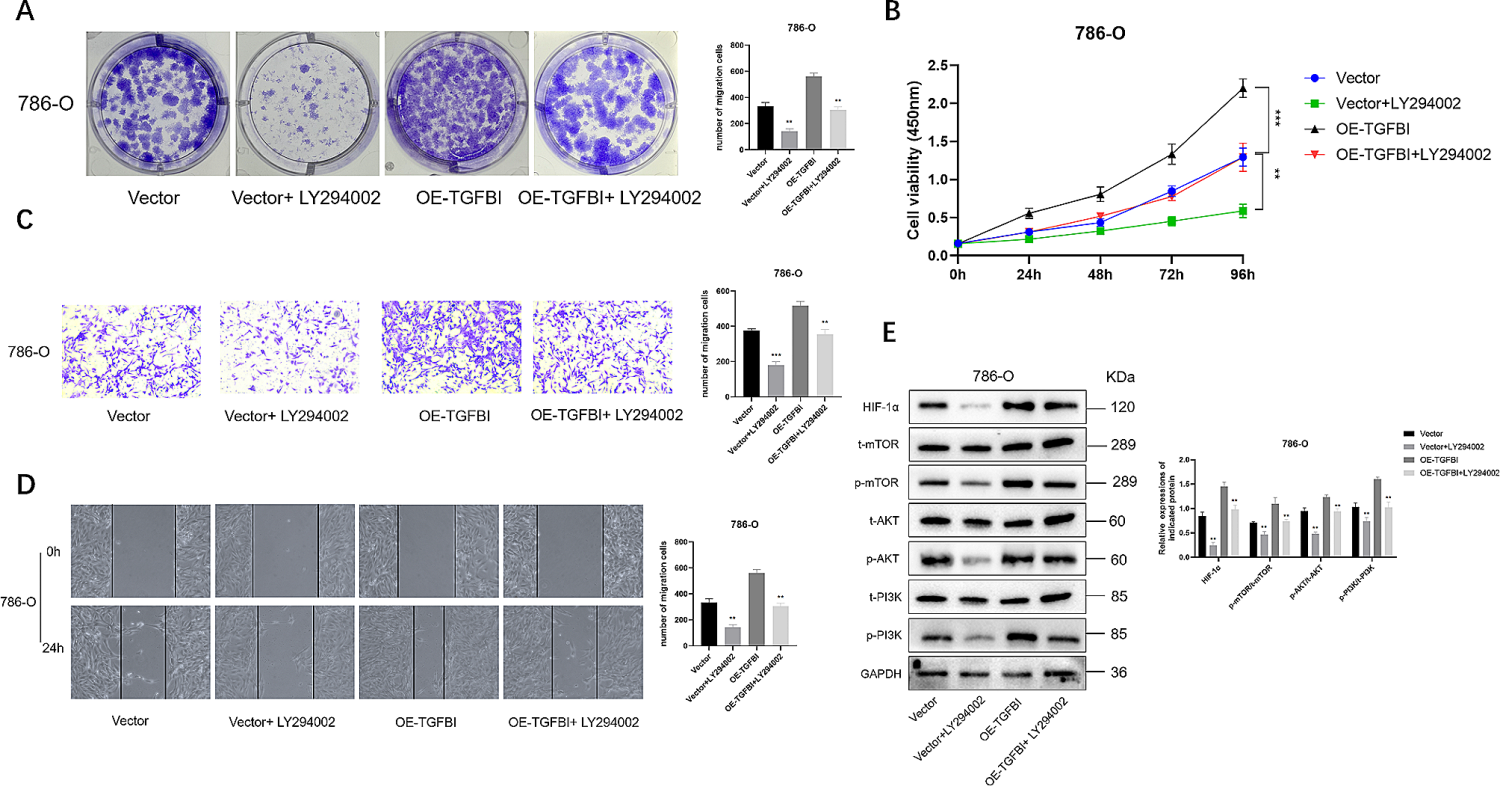
LY294002 rescues the effect of TGFBI overexpression in renal cancer cells. **(A)** Colony formation in the Vector, Vector + LY294002, OE-TGFBI, OE-TGFBI + LY294002 group in 786-O cells. **(B)** The proliferation of cells in the Vector, Vector + LY294002, OE-TGFBI, OE-TGFBI + LY294002 group at 24, 48, 72 and 96 h were detected by CCK-8 method in 786-O cells. **(C-D)** The migration and invasion of cells in the Vector, Vector + LY294002, OE-TGFBI, OE-TGFBI + LY2940029 group were detected by wound healing and transwell in 786-O cells. **(E)** Western blotting analysis of the PI3K/AKT/mToR/HIF-1α signalling pathway-related protein expression in renal cancer cells. Data are shown as mean ± SD. ** *p* < 0.01, *** *p* < 0.001compared with controls

We used the PI3K inhibitor LY294002 to treat 786-O cells transfected with pcDNA3.1-TGFBI. We found that LY294002 blocked the effects of TGFBI overexpression on cell proliferation, colony formation, migration and invasion abilities (Fig. 6A-D). Moreover, LY294002 treatment also reduced the level of HIF-1α and upregulation of AKT and mTOR phosphorylation induced by TGFBI overexpression (Fig. 6E).

### Correlation between TGFBI and immune cells and the prediction of drugs

**Fig. 7:**
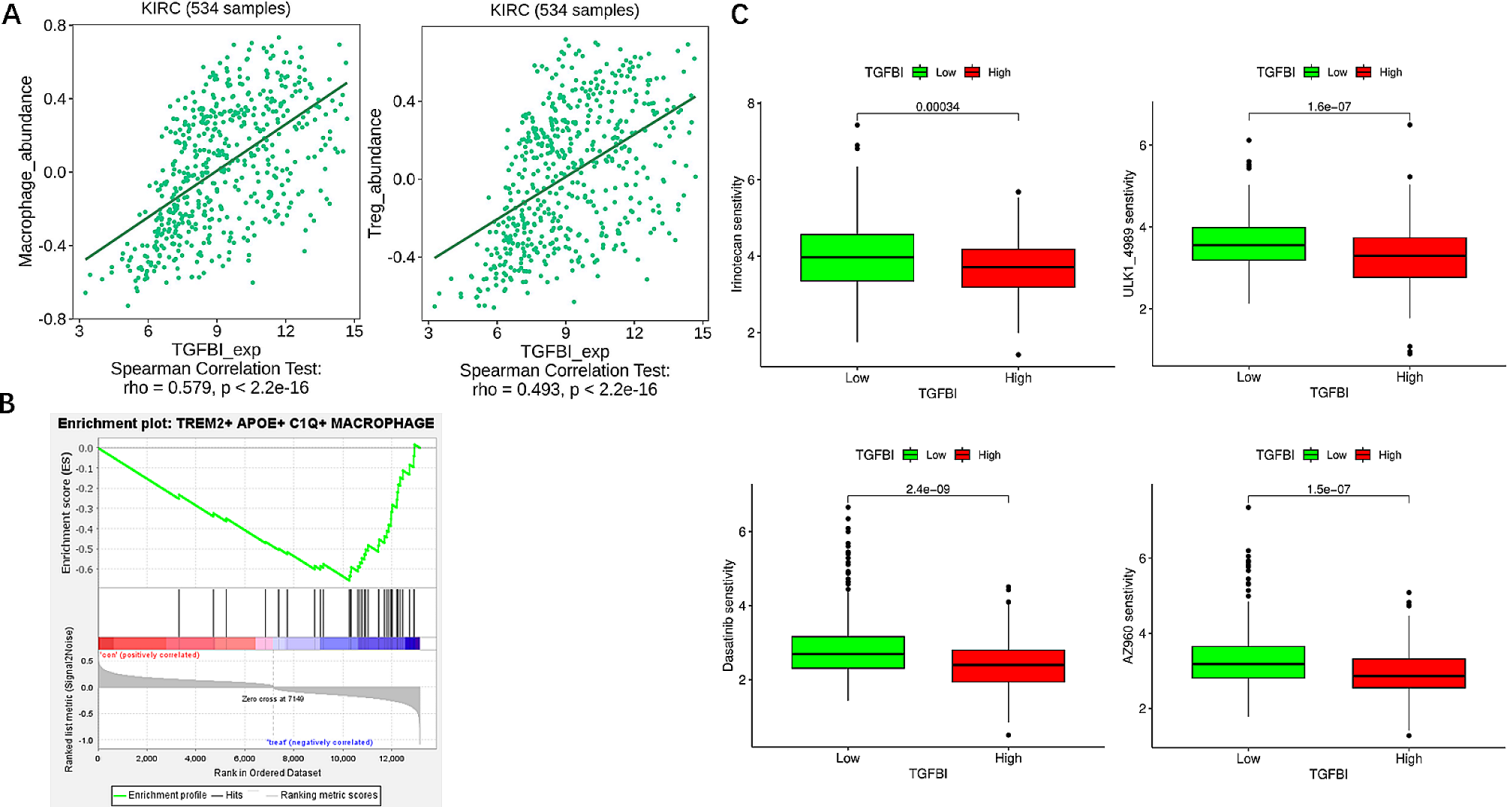
The relationship between TGFBI and the tumor immune microenvironment (TIME) and its prediction of drugs. **(A)** Among immune cells, Macrophage abundance and Treg abundance were also positively correlated with TGFBI. **(B)** TREM2^+^APOE^+^C1Q^+^Macrophages were corresponded to high TGFBI group. **(C)** Besides, Chemotherapy drugs were predicted to reveal their relationship with TGFBI expressions, drugs that had higher sensitivity in patients with higher TGFBI level were depicted

To further illustrate the role of TGFBI in the biological function of RCC and the tumor immune process, we investigated the relationship between TGFBI and immune cells, with macrophages and Treg being the top two cells connecting TGFBI expression (Fig. 7A). In our subsequent GSEA analysis, TREM2 + APOE + C1Q + macrophages could be found to be enriched in the TGFBI high-expression group (Fig. 7B), suggesting that the immune process may play a role in the disease progression of TGFBI high-expression RCC. In order to predict potential chemotherapy drug that might target TGFBI, we performed drug prediction analysis and molecular docking, The sensitivity of four drugs were found to vary dramatically from high and low TGFBI groups (Fig. 7C), we then performed molecular docking of TGFBI with these drugs (Table S4), the cavities of TGFBI which could interact with therapeutic compounds were predicted and vina scores of the cavities were calculated, commonly, lower vina score represent lower energy, which referred to stronger combination between drugs and TGFBI. Therefore, amongst all four drugs, Irinotecan seemed to possess more intimate combination with TGFBI, further experiments on animals are required for validation.

## Discussion

TGFBI is an EMC protein involved in a variety of biological processes such as EMT in tumors, and it has been shown to be an important factor involved in EMT and tumor progression processes in prostate cancer [26]. However, the involvement of TGFBI in the process of renal tumor development and its mechanism has not been clarified for the time being. Through previous studies, TGFBI gene was found to be highly expressed in renal tumor tissues [17], In our study, we also further demonstrated that TGFBI was abnormally elevated in human renal tumor tissues and various renal tumor cells, suggesting that it may be a pro-carcinogenic gene in renal cell carcinoma. To further elucidate whether TGFBI can affect the biological behavior of renal tumor cells, we designed a series of experiments to evaluate it. In our study, knockdown of TGFBI inhibited the proliferation, migration and invasion of renal cancer cells. However, the shortcoming is that we have not yet verified its effect of promoting tumour growth and invasion in in vivo experiments, and we will continue to carry out relevant studies in this part in the future.

The process of epithelial-mesenchymal transition (EMT) is accompanied by dramatic changes in cell morphology, loss and remodeling of intercellular and cellular matrix adhesion, and increased migration and invasive capacity [27]. Previous studies have shown that EMT is an important process in tumor metastasis and can lead to in situ tumor invasion of blood vessels via connecting cells [28, 29]. EMT is characterized by deletion of epithelial cell markers (e.g., cytokeratins and E-cadherin) and upregulation of the expression of mesenchymal cell markers (e.g., N-cadherin, vimentin, and fibronectin). Changes in the expression of these epithelial cell markers and mesenchymal cell markers can lead to a decrease in adhesion between transition cells and neighboring epithelial cells, which can lead to an increase in the secretion of enzymes that break down the extracellular matrix [30]. In this study, we detected some EMT-related molecules in tumor cells after knockdown TGFBI expression and found that knockdown of TGFBI reduced the expression of N-cadherin, vimentin, snail1, in addition to the expression of related fibrotic molecules (e.g., collagen3, α-SMA). Taken together, these studies may suggest that TGFBI may be an EMT-associated inducer, which may be a new target for the treatment of renal tumor metastasis.

In order to reveal how TGFBI promotes renal tumorigenesis, we used bioinformatics to discover the involvement of PI3K/AKT/mTOR and HIF-1 signaling pathway. HIF-1α is one of the key molecules in the HIF-1 signaling pathway, and previous studies have also shown that it has an important role in tumorigenesis and development [31, 32]. It has also been confirmed that the expression of HIF-1α is elevated in renal cancer tissues and is involved in renal carcinogenesis and various biological processes [33–36]. In our study, it was observed that the expression level of TGFBI was positively correlated with the expression of HIF-1α. In addition, it has been demonstrated that the PI3K/AKT/ mTOR signaling pathway is an important pathway involved in the translation of HIF-1α protein, which is consistent with our analysis. In our experiments, after knockdown of TGFBI, it was observed that TGFBI positively regulated the phosphorylation levels of PI3K, AKT, and mTOR, but did not have a significant effect on total protein expression. However, we still need further discovery and verification as to whether TGFBI regulates HIF-1α expression through other molecular mechanisms and how it specifically regulates the entire HIF-1 signaling pathway.

Tumor immune microenvironment (TIME) was regarded as an important factor that affect cancer cells proliferation and progression [37], while its majority consists of Tumor infiltrating lymphocytes (TILs) [38]. Among all TILs, Tumor associated macrophages (TAMs) were considered as a kind of cells that could induce disease progression [39]. For instance, a recent study reported a new subtype, TREM2 + APOE + C1Q + Macrophages, which was a putative marker and target for recurrent KIRC patients [40]. In our study, TGFBI was found to be correlated with macrophages and T regulatory cells. In addition, it was also shown that TGFBI is associated with TREM2 + APOE + C1Q + macrophages. Based on these results, single cell analysis revealed a remarkably higher TGFBI expression in TAMs or even cycling TAMs, so we presume that TGFBI can not only directly affect cancer cells, but also promote disease progression through TAMs, which proves its value as therapeutic targets. Based on the results from TISIDB, we found that TGFBI does not necessarily separate responders from non-responders to common immunotherapy. Therefore, we predicted drugs that might bind to TGFBI so that it could be targeted directly. Irinotecan was predicted to be the most effective drug and requires further in vivo experiments in combination with conventional immunotherapy.

## Conclusion

In summary, our study demonstrated experimentally for the first time that TGFBI is highly expressed in renal cell carcinoma, as well as facilitates renal cancer cells migration and invasion. Moreover, we further proved that TGFBI participates in the EMT process of renal cell carcinoma and affects the occurrence and development of renal cancer through the PI3K/AKT/mTOR/HIF-1α signaling pathway. In addition, it was also found for the first time that TGFBI may affect the proliferation and progression of renal cancer cells by influencing the tumor immune microenvironment. On this basis, we believe that TGFBI has great potential as a new diagnostic biomarker and therapeutic target for RCC in the future, and its molecular mechanism is worthy of further validation and investigation.

### Electronic supplementary material

Below is the link to the electronic supplementary material.

Supplementary Material 1

Supplementary Material 2

## Data Availability

No datasets were generated or analysed during the current study.

## Abbreviations

RCC: Renal cell carcinoma
qRT-PCR: quantitative real-time polymerase chain reaction
GSEA: Gene set enrichment analysis
EMT: Epithelial-mesenchymal transition
ANOVA: Analysis of variance
TCGA: The Cancer Genome Atlas
CPTAC: Clinical Proteomic Tumor Analysis Consortium
TIME: Tumor immune microenvironment
TAMs: Tumor associated macrophages
