# Supplementary material for: TGFBI promotes proliferation and epithelial–mesenchymal transition in renal cell carcinoma through PI3K/AKT/mTOR/HIF-1α pathway
Source: Cancer Cell Int. 2024 Jul 27;24:265. doi: 10.1186/s12935-024-03454-7 (PMC11282683; doi:10.1186/s12935-024-03454-7)
Supplement: Supplementary file 2 — Supplementary Material 2 [file 12935_2024_3454_MOESM2_ESM.docx]

| Gene  (Human) | Forward primer (5’~3’) | Reverse primer (5’~3’) |
| --- | --- | --- |
| ACTB | CATGTACGTTGCTATCCAGGC | CTCCTTAATGTCACGCACGAT |
| TGFBI | CACTCTCAAACCTTTACGAGACC | CGTTGCTAGGGGCGAAGATG |

**Table S1.** Primer sequences for qRT‐PCR.

**Table S2.** Primary antibodies for Western Blot and immunohistochemical.

| Antigens (Human) | Species antibodies  raised in | Dilution | Supplier |
| --- | --- | --- | --- |
| TGFBI | Rabbit, polyclonal | 1:1000 (WB)  1:200 (IHC) | Proteintech 10188-1-AP |
| Fibronectin | Rabbit, polyclonal | 1:1000 (WB) | Proteintech 15613-1-AP |
| E-cadherin  N-cadherin | Rabbit, polyclonal  Rabbit, polyclonal | 1:1000 (WB)  1:1000 (WB) | Abclonal A3044  Abclonal A21308 |
| CollageⅢ/COL3A1 | Rabbit, monoclonal | 1:1000 (WB) | Abclonal A0817 |
| Vimentin | Rabbit, monoclonal | 1:1000 (WB) | Abclonal A19607 |
| α-SMA | Rabbit, polyclonal | 1:1000 (WB) | Abclonal A7248 |
| Snail1 | Rabbit, polyclonal | 1:1000 (WB) | Proteintech 13099-1-AP |
| t-mTOR | Rabbit, polyclonal | 1:1000 (WB) | Abclonal A11354 |
| p-mTOR | Rabbit, monoclonal | 1:1000 (WB) | Abclonal AP0115 |
| t-AKT | Rabbit, monoclonal | 1:1000 (WB) | Abclonal A17909 |
| p-AKT | Rabbit, monoclonal | 1:1000 (WB) | Abclonal AP0637 |
| t-PI3K | Rabbit, monoclonal | 1:1000 (WB) | Abclonal A19684 |
| p-PI3K | Rabbit, polyclonal | 1:1000 (WB) | Abmart T40116 |
| HIF-1α | Rabbit, monoclonal | 1:1000 (WB) | Abclonal A22041 |
| GAPDH | Rabbit, polyclonal | 1:1000 (WB) | Abclonal AC001 |
| ACTB | Rabbit, monoclonal | 1:1000 (WB) | Abclonal AC038 |

**Table S3.**Secondary antibodies for Western Blot and immunohistochemical.

| Secondary detection  system used | Species antibodies  raised in | Dilution | | Supplier |
| --- | --- | --- | --- | --- |
| Anti‐Rabbit‐IgG (H+L)-HRP | Goat | | 1:10000 (WB) | Abclonal, USA, Cat.  AS014 |
| Anti‐rabbit IgG  H&L(HRP) | Goat | | 1:200 (IHC) | Abcam, USA, cat.  ab205718 |
